# Poetry

**Published:** 1881-03

**Authors:** 


### FAREWELL.

The boat went drifting, drifting, over the sleeping sea,
And the man that I loved the dearest sat in the boat with me.

The shadow of coming parting hung over the great gray swell,
And the winds that swept across it sobbed on farewell, farewell.

The boat went drifting, drifting, in the lingering northern night,
And the face that I loved the dearest paled with the paling light.

We strove to join light laughter; we strove to wake a jest;
But the voice that I loved the dearest rang sadly 'mid the rest.

The boat went drifting, drifting, while the dull skies lowered down,
And the "ragged rims of thunder" gave the rocky head a crown.

The boat went drifting, drifting, while to the darkening sky,
For the man that I loved the dearest, the prayer rose silently.

Oh, true, strong hand I touch no more; brave smile I may not see;
Will the God who governs time and tide bring him back to my life and me?

—*All the Year Round.*

### A CHARACTER SKETCH.

The mule seemed pensive, even sad,
As if by conscience pricked:
But, when they came to share his woes,
He raised objections—kicked.

The cat came up to sympathize,
With mew and gentle purr;
Alas! she got within his reach,
When—fiddlestrings and fur.

The dog, in pity, neared him, to
Alleviate his care;
He tried to pass around him once,
But—sausage-meat and hair.

And John, the honest farmer-boy,
Who had the beast in charge,
Tried recklessly to harness him—
His funeral was large.

Oh, trifling were the causes which
His flexile legs unfurled;
And many were the quadrupeds
That sought another world.

He never did a decent thing,
He wasn't worth a ducat;
He kicked and kicked until he died
And then he kicked the bucket

### REJOICE.

Burden not thy soul with sadness;
Make a wiser, better choice;
Drink the wine of life with gladness;
God doth bid thee, man, "rejoice!"
In to-day's bright sunlight basking,
Leave to-morrow's cares alone;
Spoil not present joys by asking
"Who shall roll away the stone?"

### IMPERFECTUS.

I wonder if ever a song was sung
But the singer's heart sang sweeter!
I wonder if ever a rhyme was rung
But the thought surpassed the meter!
I wonder if ever a sculptor wrought
Till the cold stone echoed his ardent thought!
Or if ever a painter, with light and shade,
The dream of his inmost heart portrayed!

I wonder if ever a rose was found,
And there might not be a fairer!
Or if ever a glittering gem was ground,
And we dreamed not of a rarer!
Ah! never on earth shall we find the best!
But it waits for us in the Land of Rest:
And a perfect thing we shall never behold
Till we pass the portal of shining gold.

—*J. C. Harney in Independent.*

